# Mutational signatures in 175 Chinese gastric cancer patients

**DOI:** 10.1186/s12885-024-12968-2

**Published:** 2024-09-30

**Authors:** Fatao Liu, Nan Hu, Kewei Jiang, Huaitian Liu, Mingyi Wang, Ying Hu, Tongwu Zhang, Ho-Hsiang Wu, Howard Yang, Hao Weng, Ping Dong, Carol Giffen, Bin Zhu, Maxwell P. Lee, Christian C. Abnet, Philip R. Taylor, Yun Liu, Yingbin Liu, Alisa M. Goldstein

**Affiliations:** 1 Shanghai Key Laboratory of Biliary Tract Disease Research, Shanghai, 200092 P. R. China; 2 Department of General Surgery, Xinhua Hospital, Shanghai JiaoTong University School of Medicine, Shanghai, 200092 P. R. China; 3 Division of Cancer Epidemiology and Genetics, National Cancer Institute, Bethesda, MD 20892 USA; 4 Department of Gastrointestinal Surgery, Peking University People’s Hospital, Beijing, 100044 P. R. China; 5 Center for Cancer Research, National Cancer Institute, Bethesda, MD 20892 USA; 6 Information Management Services, Inc, Silver Spring, Bethesda, MD 20904 USA; 7 Department of Oncology, Fudan University Pudong Medical Center, Shanghai, P. R. China

**Keywords:** Gastric cancer, Somatic alterations, Mutational signatures, Driver genes, Tumor molecular heterogeneity

## Abstract

**Background:**

Gastric cancer (GC), a molecularly heterogeneous disease, is the third leading cause of cancer death worldwide. The majority of GC cases worldwide occur in East Asia, predominantly China. Mutational Signature Framework offers an elegant approach to identify mutational processes present in tumors.

**Methods:**

To identify mutational signature patterns, we conducted whole exome sequencing (WES) analysis in Chinese patients with GC. Mutect2 and MutsigCV were used to identify significantly mutated genes in 175 Chinese GC cases using paired tumor-normal tissues. We investigated mutational signatures using Catalogue of Somatic Mutations in Cancer (COSMIC) Version 2 (V2) and Version 3 (V3).

**Results:**

We identified 104 mutated genes with *P* < 0.01. Seven genes (*OR6B1*,* B2M*,* ELF3*,* RHOA*,* RPL22*, *TP53*, *ARIDIA)* had q < 0.0001, including six previously associated with GC. Mutational signatures (COSMIC-V3) observed include 14 single base substitutions (SBS), one doublet base substitution (DBS) Signature A, and one InDel (ID2). The most frequent SBS signatures (SBS05, SBS01, SBS15, SBS20, SBS40) were also observed in 254 White GC cases from The Cancer Genome Atlas (TCGA) Project. However, SBS01 and SBS20 showed significant differences between Whites vs. All Asians (19.3% vs. 11.3% for SBS 1 (*P* = 0.012) and 11.4% vs. 5.9% for SBS20 (*P* = 0.025), respectively). Using COSMIC V2, signatures 6, 15, and 1 were the most frequent in Chinese GC cases. Further, most Chinese GC cases carried multiple signatures.

**Conclusions:**

This effort represents the most detailed mutational signatures analysis of GC cases from China to date. Results hold promise for new insights in understanding risk and prognosis factors in GC.

**Supplementary Information:**

The online version contains supplementary material available at 10.1186/s12885-024-12968-2.

## Background

Gastric cancer (GC) is the third leading cause of cancer death worldwide. It is a highly heterogeneous disease with multiple causes although *Helicobacter pylori* is a major causative agent, particularly for distal GC [1–3]. More than half of GC cases worldwide occur in East Asia, predominantly in China [4].

Recently, using next generation sequencing (whole-genome sequencing (WGS) and whole exome sequencing (WES)), the Cancer Genome Atlas Network (TCGA) and the Asian Cancer Research Group (ACRG) identified several sub-types of GC based on genomic molecular alterations and tried to decipher the clinical and molecular heterogeneity of GC to target therapies for this disease. TCGA identified four subtypes of GC: Epstein-Barr virus positive (EBV^+^), microsatellite instability (MSI), genomically stable (GS), and chromosomal instability (CIN) based on 295 GC cases that included 75% White and 25% Asian patients [5]. ACRG also proposed four molecular subtypes of GC: MSI, microsatellite stable and epithelial-to-mesenchymal transition (MSS/EMT), MSS/TP53^−^, and MSS/TP53^+^, based on whole genome sequencing (WGS) of 49 GC tumors and gene expression and copy number profiling as well as targeted gene sequencing of 251 GC tumors from Korean patients [6].

GC also shows histological variability and several classifications have been proposed, but the most frequently used systems are the Lauren system [7] and the WHO classification [8]. The WHO classification is based on the major histological patterns of tubular, papillary, mucinous, and signet-ring cell carcinoma [8]. Anatomically, GC is also classified as ‘cardia’ or ‘noncardia’ based on its location in the stomach.

Since 2001, WES and/or WGS results have been reported from more than 1000 GC cases [5, 9–28]. From these studies, several previously identified significantly mutated genes were confirmed in GC cases from Asia, including *TP53*,* CHD1*,* RHOA*,* PIK3CA*, and *ARID1A* [6, 9, 10, 25–27]. However, results from these studies do not fully explain the heterogeneity of clinical and molecular alterations in GC from Asia. Since most GC cases from Asia occur in China, we aimed to reduce the heterogeneity among Asian GC cases overall by focusing on molecular changes in Chinese GC patients. In addition, we expanded the evaluation of sequencing data to include mutational signatures. In the past few years, mutational signature analyses have shown diagnostic or therapeutic value in numerous etiologically distinct subgroups of tumors [29–34]. Additionally, mutational signatures have been associated with several risk factors, including tobacco [35, 36]. Among the previously published WES/WGS papers on Asian GC cases, the numbers of Chinese patients were relatively small [9, 11, 14, 17, 19, 20, 24, 28] and most of these studies focused on discovery of driver and GC-related genes. Three of these studies investigated profiles of the six base substitutions [19, 20, 24], but no analyses evaluated all 96 possible nucleotide base substitutions, and only two studies attempted to identify mutational signatures [26, 27]. Chen et al. observed mutational signatures 1, 6, and 17 from WES of 74 Chinese GC cases, coupled with evaluation of two publicly available WES datasets [26]. Based on WGS in 168 GC cases, Xing et al. identified six signatures, including signatures 14*, 17*, and 18*, and three “unmatched” signatures related to age, APOBEC, and DNA mismatch repair (MMR) deficiency [27]. However, neither study provided full details of the distribution or frequency of mutational signatures.

The Wellcome Trust Sanger Institute (WTSI) Mutational Signature Framework offers a comprehensive approach to identify signatures of mutational processes in tumor samples and then determine the contribution of each mutational process to each individual sample using the Catalogue of Somatic Mutation In Cancer (COSMIC) signature website [37]. COSMIC version 2 (V2-March 2015) includes 30 mutational signatures (http://cancer.sanger.ac.uk/cosmic/signatures) [37]. Accurate deconvolution of signatures, however, requires a large sample size. For smaller sample sizes, other tools are available and may be used to identify mutational signatures, for example, modified DeconstructSigs [38].

Some signatures may involve multiple mutational processes and the catalogue of mutational signatures continues to incorporate new knowledge. Alexandrov et al. [39] updated the numbers and types of mutational signatures based on evaluation of 23,829 samples (19,184 with WES and 4645 with WGS) that included most cancer types, including more than 400 GC cases, but only limited numbers of Asian GC cases (*n* = 81). This update recharacterized 67 single base substitution (SBS) signatures (based on 96 classes determined by the six base substitutions), 11 doublet base substitution (DBS) signatures (based on 78 classes of doublet base substitutions), four clustered base substitution signatures, and 17 small insertion and deletion (ID) mutational signatures (based on 83 classes according to the size of the indel, repeat, and microhomology) [39]. Based on this extensive re-evaluation, COSMIC released mutational signature Version 3 (V3-May 2019). A subset of 19 SBS signatures, however, have recently been suggested to be possible sequencing artifacts [SBS27, SBS43, SBS45-SBS60, SBS95] [http://cancer.sanger.ac.uk/cosmic/signatures] [37].

The present study aimed to identify molecular alterations in 175 Chinese GC cases through WES of their tumors, and to examine the distributions of mutational signatures in these tumors to evaluate the more recent mutational signatures (V3) and to compare results to previous investigations that used COSMIC-V2 [37].

## Methods

### Patient enrollment, clinical data collection, and DNA extraction

Samples from 183 surgical patients with pathologically confirmed gastric cancer were collected from 2011 to 2015 at the Department of General Surgery, Xinhua Hospital, School of Medicine, Shanghai Jiaotong University, China. None of the patients received chemotherapy or radiotherapy before surgery. Fresh tissue was processed within 15 min after removal. Each sample was frozen and stored at − 80 degrees C. Paired non-cancerous tissues were dissected at least 2 cm away from the tumor border and were confirmed to lack tumor cells by microscopy. For the tumor tissue, tumor cells comprised at least 70% of the tumor. For immunohistochemical staining, tissues were fixed in 4% formalin immediately after removal and embedded in paraffin. All samples were confirmed by pathological diagnosis and staged according to the 7th AJCC-TNM Classification (American Joint Committee on Cancer) of Malignant Tumors [40] by pathologists in Xinhua Hospital, Shanghai, China. This study was approved by the ethics committee of Xinhua Hospital (XHEC-D-2017-038) and written informed consent was obtained from all participants before enrollment.

Tumors and corresponding non-cancerous tissues were ground in liquid nitrogen. DNA was extracted and purified using QIAamp DNA kits (Qiagen). A Qubit 2.0 fluorometer (Life Technologies, Carlsbad, CA) and a Thermo NanoDrop 2000 (Thermo, Wilmington, DE, USA) were used to determine DNA concentration and quality. The integrity of DNA was also confirmed by agarose gel electrophoresis.

We combined mucinous adenocarcinoma (*n* = 27) and signet ring cells (*n* = 9) and called this group non-tubular GC. Cases with mixed tubular and non-tubular histology were classified as non-tubular. Well differentiated (WD) (*n* = 4) and moderately differentiated (MD) tumors were combined into MD, and cases with both MD and poorly differentiated (PD) features were classified as PD.

### WES library preparation, exome capture, and sequencing

The WES library was established following Illumina protocols (TruSeq DNA Sample Prep Kit). Briefly, the libraries were constructed from 500 ng of sheared genomic DNA following purifying, end repair, 3’ end adenylation, indexed pair-end adaptors ligation, ligation products purification and PCR amplification. The products were then subjected to exonic hybrid capture using either the TruSeq Exome Enrichment kit (Illumina) or SeqCap EZ capture kit (Roche). For TruSeq, after library validation, normalization and pooling, the libraries were sequenced with the Illumina HiSeq 2500 Genome Analyzer, yielding 200 (2 × 100) base pairs from the final library fragments. For SeqCap EZ, after library validation, normalization and pooling, the libraries were sequenced with the Illumina HiSeq X Ten platform, yielding 300 (2 × 150) base pairs from the final library fragments. Sequencing depth was 100x for tumors and 50x for corresponding non-cancerous tissues.

### Data analysis

#### Sequence analysis

Quality-based trimming and filtering using Trimmomatic (version 0.30) [41] were conducted on paired-end reads from 183 paired tumor and matched normal samples. In this step, we removed the Illumina-specific adapter and other low-quality sequences from the reads. We removed the leading and trailing bases in a read if the quality scores were below 3 and scanned from 5’ end of the read with a 4-base sliding window. We also removed the 3’ end of the read when the average quality per base dropped below 15. We also removed reads less than 36 bases after trimming. Next, sequence data were aligned to hg19 version of the human reference using BWA (version 0.6.2) [42] and deduplicated by Picard [43]. Local realignment around suspected indels sites and base quality recalibration was performed using Genome Analysis Toolkit (GATK) IndelRealigner (GATK version 3.1-1) [44].

#### Quality control

We ran FastQC [45], BAM-matcher [46], and Picard [43] to collect quality control metrics before and after the alignment workflow to remove low-quality samples. Based on quality control metrics, we removed eight low quality sample pairs with high oxidation error rates. Thus, 175 sample pairs were retained for analysis. Sequencing coverage and quality control statistics for each sample are summarized in Additional file 1 and are based on GATKDepthOfCoverage version 4.1.6.0 [44]. These statistics include the total number of sequenced reads, uniquely mapped nonduplicate reads, and covered bases, mean coverage per base, and percentage of targeted bases with coverage ≥ 10. Whole exome sequencing data may be found at:

https://dataview.ncbi.nlm.nih.gov/object/PRJNA702785?reviewer=jsvnkpti6epj03pf7l1o1ugimp.

#### Variant calling, somatic mutation calling and mutated gene selection

Before somatic variant calling, we generated a Panel of Normals (PONs) for all 175 normal samples using GATK Mutect2 (v3.4-0) [44, 47] to filter out germline variants and other artifacts. Then, somatic single nucleotide variants (SNV) and small insertions and deletions for each pair were identified using Mutect2 [47] with default parameters plus the PONs filter. All high-confidence mutations were obtained using an in-house pipeline coupled with visual inspection, then annotated with Ensemble VEP (version 75) [48], and converted into Mutation Annotation Format (MAF) files using vcf2maf [49]. Since this study was focused on somatic mutations, we did not examine the sequenced data for germline alterations.

Potential significantly mutated genes were further evaluated using MutsigCV (v1.4.1) [50]. For this analysis, *P* < 0.01 was considered significant. A False Discovery Rate (q-value) was also calculated; to be conservative, genes with q < 0.01 were considered significantly mutated.

### Mutational signatures

#### Evaluation using COSMIC V3 mutational signatures

Mutational signatures were extracted based on the final mutation calling sets using the previously developed computational framework SigProfiler [29]. A detailed description of the workflow of the framework has also been published [39], and the code can be downloaded freely from (https://www.mathworks.com/matlabcentral/fileexchange/38724-sigprofiler*).*

For comparison purposes, we analyzed SBS signatures for 335 GC samples from TCGA and compared 254 White TCGA GC samples to 256 All Asian GC samples (81 GC from TCGA and 175 in the current study). For the comparison of frequencies based on the observation of a SBS signature for the 254 White TCGA GC samples versus the 256 Asian GC samples, as is customary, we considered a signature as being absent from a sample if the number of mutations attributed to that signature was less than 100. This approach helps mitigate overfitting issues associated with the uncertainty arising from a limited number of mutations assigned to a specific signature. For the TCGA data, the mutational signatures previously determined using SigProfiler [29] based on WES data were extracted from https://www.synapse.org/#!Synapse:syn11726601. (file name: TCGA_WES_sigProfiler_SBS_signatures_in_samples.csv). We extracted the TCGA Stomach cancer samples using cancer type “Stomach-AdenoCa”. For both datasets, mutation signatures with frequencies < 0.6% were set to zero to reduce overfitting. All mutation signature figures were generated with R ggplot2 package [51].

#### Evaluation using COSMIC V2 mutational signatures (without DBS and InDels)

Since previous evaluations of mutation signatures in Chinese GC patients used COSMIC V2, we used the following strategy for evaluation of mutational signatures results from COSMIC v2 [37]. The Sequence Context (TSC) immediately 3′ and 5′ of the single nucleotide mutated base called by MuTect2 was extracted by bedtools (v.2.27.1) [52] and an in-house Linux awk script. To identify mutational signatures using COSMIC V2, we used two approaches. First, we used generalized linear models (GLM) as follows:

$$\:\left(\text{G}\text{L}\text{M}\right)\varvec{fit}=\text{glm}\left({sig.ref}_{i}\sim{sig.ind}_{j}\right)$$

$$\:\varvec{s}\varvec{u}\varvec{m}\:=\:\text{r}\text{b}\text{i}\text{n}\text{d}\left(\varvec{f}\varvec{i}\varvec{t}\right)$$

where for i = 1 to M, and j = 1 to N, M is the set of 30 COSMIC mutational signatures, and N is the number of cases (*N* = 175). We used a predetermined arbitrary cut-off of -log10 *P*-value > 6 (*P* < 1.00E-06). If an individual TSC was mapped to multiple signatures, the signature with the maximum -log10 *P*-value was selected. We performed non-negative matrix factorization (NMF) to examine signature heterogeneity in the 175 GCs. For TSCs that could not be mapped to any COSMIC signature, we clustered cases separately by their trinucleotide sequence contexts using hierarchical clustering. To evaluate consistency of the mutational signature findings based on COSMIC V2, we used a second method with a different statistical framework: a modified version of deconstructSigs (modified deconstructSigs) [38] in R.

For modified deconstructSigs [38], we used the BSgenome.Hsapiens.UCSC.hg19 reference, the signatures.cosmic comparison set, a signature cutoff value of 0.02, and a tri.counts.method parameter of “default.” To reduce over-fitting of previously identified consensus signatures of mutation processes, we evaluated the signature contribution in each sample by minimizing the cosine similarity between the original sample and reconstructed sample as previously reported [53]. Overall, any mutational signature contributing a low proportion of somatic mutations in a sample was removed, and the sample was re-analyzed with the remaining signatures. In addition, any signature that did not improve the cosine similarity between the original sample and the sample reconstructed with the consensus mutational signatures, and their respective exposures with > 0.02, was removed. The sample was then re-analyzed with the remaining signatures. This method allowed us to detect each of the mutational signatures that significantly contributed to the mutations in each sample. Since these methods are based on different statistical frameworks, some differences in results are to be expected. Our strategy for these comparisons was to compare the trends in the same signature patterns in GCs across the two methods. We restricted the presentation of mutation signatures to those that were observed in greater than 2% of samples.

## Results

We conducted WES on DNAs from matched tumor and normal tissues isolated from 175 Chinese individuals with GC. The clinical data for the GC patients is shown in Additional file 2. In brief, the median age of cases at diagnosis was 64 years (range 27–90 years) and males predominated (73%). Clinically, 80% of tumors were in the non-cardia and 18% in the cardia, and 59% had evidence of lymph node metastasis. 79% of tumors had tubular histology and 73% were poorly differentiated. Nearly 62% of patients were alive at follow-up, which concluded on October 31, 2016.

### Mutated genes

The type and number of somatic alterations for each case is shown in Additional file 3. Overall, 20,970 variants were detected (median = 66/case, range = 1-1949/case), including 16,541 missense variants, 986 nonsense variants, 425 splicing alterations, and 2987 InDels (2435 deletions and 552 insertions) using Mutect2. The average alterations/per Mb was 1.87 (range: 0.02–30.45). The top 5% of cases (*n* = 9) showed much higher somatic alterations than the remaining cases (670–1949 variants/case vs. 1-371 variants/case, respectively); in addition, the bottom 10% of cases (*n* = 18) had fewer than 10 somatic alterations (Additional file 3), denoting heterogeneity at the genomic level in these tumors.

Ninety-five genes had a mutation frequency of ≥ 5%. From MutSigCV, 104 mutated genes had *P* < 0.01 (Additional file 4) but only seven were considered significant based on a q-value < 0.01 (*ARID1A*,* B2M*, *ELF3*, *OR6B1*, *RHOA*, *RPL22*, and *TP53* (Table 1**).** In these seven significantly mutated genes, the proportion of mutated cases with non-cardia cancer is as expected, based on the percentage of cases with non-cardia cancer in the dataset. This distribution (i.e., proportion of mutated samples in these 7 genes) was also as expected for histology and differentiation status. Six of these genes (*ARID1A*,* B2M*,* ELF3*,* RHOA*,* RPL22*, and *TP53)* were observed in at least 2% of patients, but only *ARID1A* and *TP53* were observed in more than 10% of patients (Table 1). All six of these genes have previously been shown to be mutated in GC.

**Table 1:** Type and number of alterations in 7 significantly mutated genes by MutSigCV analysis (q < 0.01; *n* = 175 GC cases)*

No.	Gene	Frame_Shift _Del	Frame_Shift _Ins	In_Frame _Del	In_Frame _Ins	Missense _Mutation	Nonsense _Mutation	Nonstop _Mutation	Splice _Site	Translation _Start_Site	Total alterations	Altered Samples	*P* value	q value	Frequency of alterations in 175 GC cases
1	*OR6B1*	1	0	0	1	1	0	0	0	0	3	2	0	0	0.010
2	*B2M*	5	1	0	0	0	0	0	0	0	6	5	0	0	0.030
3	*RPL22*	6	0	0	0	0	0	0	0	0	6	6	0	0	0.034
4	*ELF3*	1	1	1	0	3	0	1	0	0	7	7	0	0	0.040
5	*RHOA*	1	0	0	0	7	0	0	0	0	8	8	0	0	0.046
6	*ARID1A*	10	3	0	0	3	8	0	0	0	24	20	1.89E-15	5.09E-12	0.114
7	*TP53*	5	3	0	1	44	4	0	5	0	62	61	9.99E-16	3.14E-12	0.359

*Genes ordered by frequency of alterations in GC cases from lowest to highest

Abbreviations: GC, gastric cancer; Del, deletion; Ins, insertion

**Table 2:** COSMIC V2 Mutational signatures based on single base substitutions. COSMIC V2 mutational signature frequency distribution based on all single base changes in GC cases by GLM model (*P* < 1.00E-06); *n* = 132 GC cases)*

Signature	No. of cases (frequency) by signature (*n* = 132 GC cases with 421 total signatures)^
Signature 6	113 (0.86)
Signature 15	108 (0.82)
Signature 1	99 (0.75)
Signature 14	80 (0.61)
Signature 19	8 (0.06)
Signature 17	6 (0.05)
Signature 20	2 (0.02)
Signature 4	1 (0.01)
Signature 10	1 (0.01)
Signature 23	1 (0.01)
Signature 26	1 (0.01)
Signature 29	1 (0.01)

*Cases designated as “NoMapping” (*n* = 43) were excluded

^The number includes all significant signatures since most cases carried more than two significant signatures. For details of each significant signature by case, see Additional file 11

### Characterization of somatic single nucleotide substitutions and mutational signatures in 175 GC

As expected, the rate of transitions (Ti) was higher than transversions (Tv) (55.1% vs. 44.9%). Frequencies of the six types of base substitutions were C > T (42.0%), C > A (24.8%), T > C (13.6%), T > G (8.0%), T > A (6.0%) and C > G (6.0%), including all variants (mutations and InDels) (Additional file 5). The distribution and summary distribution frequency of 96 trinucleotide changes in the 175 GC cases are shown in Additional file 6.

### COSMIC V3 mutational signatures

In the 175 GC patients, we observed 14 SBS signatures with cutoff above 0.57% (corresponding to at least one out of 175 cases), one DBS signature A that did not match any of the 11 COSMIC DBS signatures, and three ID signatures.

**Fig. 1:**
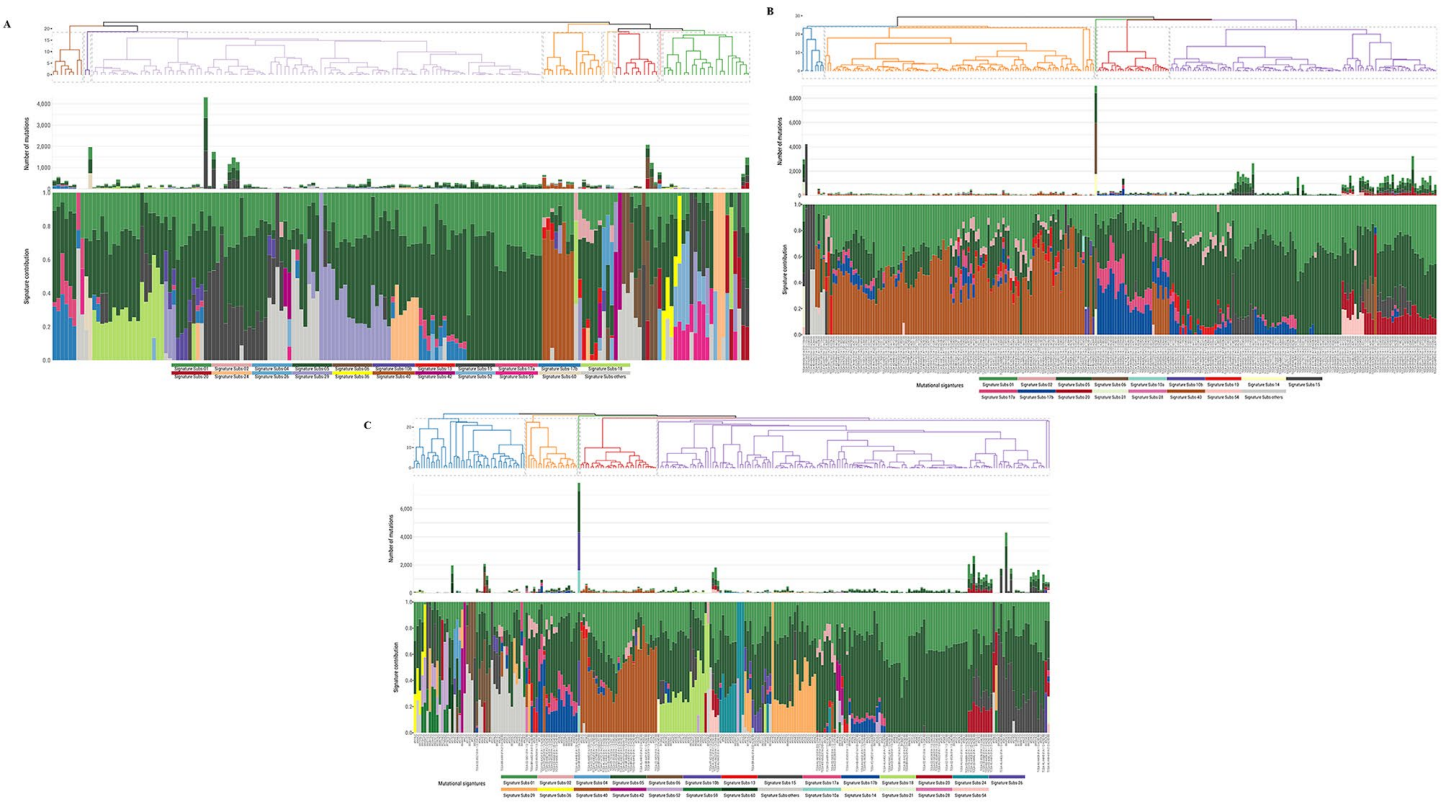
Clustering of SBS signatures using COSMIC V3 in three groups. Top panel shows clustering based on the distribution of mutational signatures for each case; middle panel shows the number of mutations assigned to each signature by case; bottom panel shows the proportion or distribution of each signature by case. (**A**) Distribution of SBS mutational signatures in current Chinese GC study (COSMIC V3; *n* = 175 cases). (**B**) Distribution of SBS mutational signatures in White GC cases from TCGA (COSMIC V3; *n* = 254 cases). (**C**) Distribution of SBS signatures in ALL Asian GC, including current Chinese GC study and Asian GC in TCGA (COSMIC V3; *n* = 256 cases)

Figure 1A and Additional file 7 show the SBS signatures for the 175 GC patients. Based on the absolute number of mutations, SBS05 showed the highest frequency (38.5%) followed by SBS01 (22.1%), SBS15 (12.1%), SBS06 (4.3%), and SBS40 (4.1%). Most cases had multiple SBS signatures: 148 cases carried ≥ 3 SBS signatures, 25 cases carried two SBS signatures, and two cases carried one SBS signature.

**Fig. 2:**
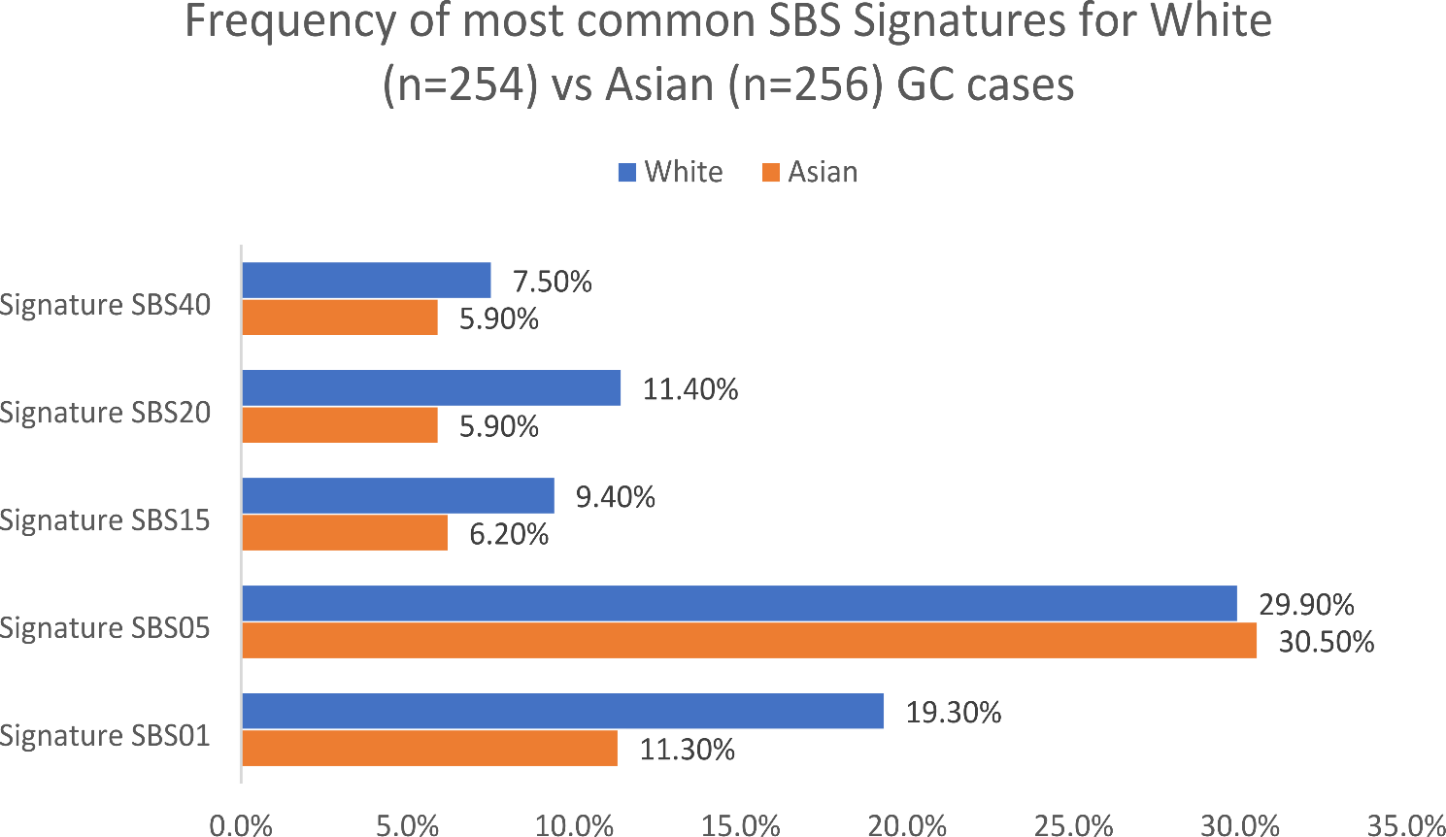
Frequency of five most common SBS mutational signatures. Comparison of common SBS mutational signatures in 256 ALL Asian GC cases (175 from current Chinese study and 81 from TCGA) compared to 254 White GC cases from TCGA (COSMIC V3)

Comprehensive landscapes of the SBS mutational signatures in 254 White GC cases from TCGA (Fig. 1B) and 256 ALL Asian (175 cases from the current study and 81 Asian cases from TCGA) GC cases (Fig. 1C**)** show the relative proportion of signatures by GC case (bottom panel) and the absolute number of mutations assigned to each signature by GC case (middle panel). Figure 2 shows the frequencies for the five most common SBS signatures in White and All Asian GC cases. For this figure and subsequent evaluation, a case is considered to have a particular signature only when there are ≥ 100 mutations attributed to a given signature, to mitigate overfitting issues (see Methods). In general, the SBS signature frequencies in the White versus all Asian GC cases were similar across most signatures, however, SBS01 and SBS20 showed significant differences [White vs. All Asian were 19.3% vs. 11.3% for SBS01 (*P* = 0.012) and 11.4% vs. 5.9% for SBS20 (*P* = 0.025)]. Because of the association between SBS20 signatures and *POLD1* mutations [37, 39], we checked the sample sets for *POLD1* mutations and observed the following: 10 White GC cases with somatic *POLD1* mutations, half of which occurred in cases with SBS20. For the Asian GC cases, there were 6 cases with somatic *POLD1* mutations, only 1 of which had SBS20.

Examination of DBS signatures revealed only a single signature “A” that did not fit any of the DBS signatures based on COSMIC V3. The number of signature A for DBS for each case and the distribution of the 78 classes of DBS for the “unmatched” DBS signature A are shown in Additional file 8.

Of the three ID signatures observed, only signature A matched a known COSMIC ID signature, signature ID2, with similarity (percent pattern overlap) of 0.97. Almost all (*n* = 171) Chinese GC cases showed ID2 (with mutation number range of 1-3977), where 12 cases had mutation number > 100 of ID2. In contrast, signatures B (88 cases with mutation number range 1–92) and C (61 cases with mutation number range 3–40) did not match any ID signature. The details of the ID signatures are shown in Additional files 9 and 10.

### COSMIC V2 mutational signatures (without DBS and InDels)

**Fig. 3:**
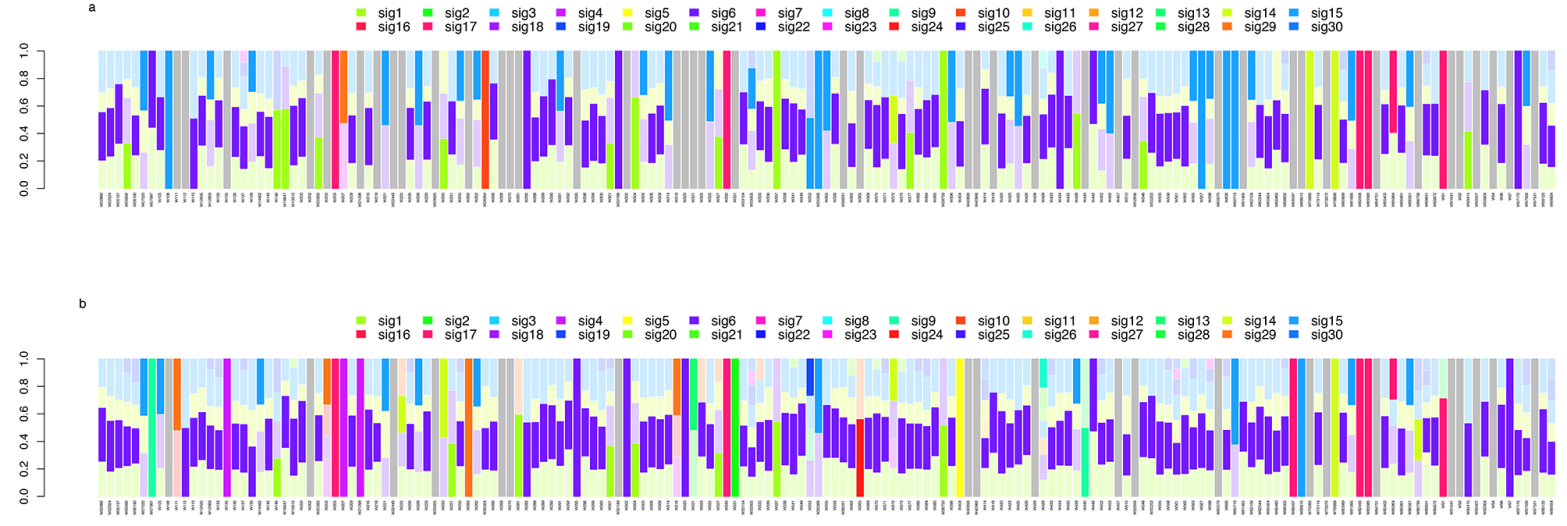
Distribution of mutational signatures using COSMIC V2. (**A**) Distribution of mutational signatures based on all single base changes (“ALL”) (COSMIC V2); 43 cases did not map to V2 signatures (gray bar). (**B**) Distribution of mutational signatures based on single base changes with amino acid alterations only (“AA”) (COSMIC V2); 21 cases did not map to V2 signatures (gray bar)

As mentioned in the Methods section, previous studies on GC used COSMIC V2. Therefore, to compare our results with previous studies [26, 27], we examined COSMIC signatures using V2 based on single base alterations only (without DBS and InDels) using GLM models. We investigated all single base changes (“ALL”) plus a subset focused on single base changes that cause amino acid alterations (“AA”) to examine relationships between signatures and mutated genes with functional alterations in GC tumors. Using COSMIC V2 and *P* < 1.00E-06, tumors in 132 of 175 GC cases matched at least one mutational signature in “ALL” (Table 2 and Additional file 11). The most frequent significant signatures in these 132 GC cases were 6, 15, 1, and 14 (present in 113, 108, 99, and 80 cases, respectively). Most cases carried at least two significant signatures (Additional file 11). Figure 3A shows the distribution of COSMIC signatures based on “ALL” in each of the 175 GC. When we calculated the frequency distribution of the single most significant COSMIC V2 mutational signature for each case, signature 6 was the most frequent significant signature, identified in 77 cases, while signature 15 was seen in 30 cases, and signature 1 in 14 cases (Table 3).

**Table 3:** COSMIC V2 Mutational signatures based on single base substitutions. Frequency distribution of the single most significant COSMIC V2 mutational signature for each case

Signature	No. of cases (frequency) by signature based on all single base changes	No. of cases (frequency) by signature restricted to amino acid altering base changes
Signature 6	77 (0.44)	107 (0.61)
Signature 15	30 (0.17)	12 (0.07)
Signature 1	14 (0.08)	8 (0.05)
Signature 17	6 (0.03)	7 (0.04)
Signature 14	3 (0.02)	5 (0.03)
Signature 10	1 (0.01)	0 (0.00)
Signature 29	1 (0.01)	4 (0.02)
Signature 4	0 (0.00)	3 (0.02)
Signature 9	0 (0.00)	2 (0.01)
Signature 13	0 (0.00)	1 (0.01)
Signature 19	0 (0.00)	1 (0.01)
Signature 29	0 (0.00)	1 (0.01)
Signature 24	0 (0.00)	1 (0.01)
Signature 26	0 (0.00)	1 (0.01)
Signature 5	0 (0.00)	1 (0.01)
NoMapping	43 (0.25)	21 (0.12)
TOTAL	175	175

When we restricted the evaluation to “AA”, we were able to map signatures in all but 21 of the 175 GC cases (Table 3 and Fig. 3B). Similar distributions of signatures were observed for “AA” compared to “ALL”, albeit at different frequencies (Additional file 12).

We used modified deconstructSigs to evaluate the robustness of our findings from the mutational signatures GLM “AA” analysis. The top signatures identified using modified deconstructSigs were signatures 6 (55%), 15 (29%), 3 (25%), 1 (16%), and 4 (11%) (Additional files 13 and 14).

Although there was variation in the frequencies for the top mutation signatures for the two statistical methods used, the rank order for the top signatures was consistent. Signature 6 was ranked first, followed by signature 15 for both methods; signature 1 was one of the top three ranked signatures for both methods. We noted that signature 3 was the third ranked signature for the modified deconstructSigs analysis but was not observed in GLM. This discordance may reflect differences in the statistical framework whereby the GLM method selects only the most significant signature in each case.

Because signature 6 has been associated with mismatch repair genes [37], we searched for mutations in the major MMR genes including *MLH1*, *MSH2*,* MSH6*, *PMS1*, and *PMS2*. *MSH6* had two frameshift deletions and two missense mutations in 3 cases; *MSH2* and *PMS2* each had one missense mutation from the same case (W562) which also had the two frameshift deletions in *MSH6.* The three cases with these MMR gene AA mutations showed COSMIC V2 signature 6.

Signature 6 is also associated with small (shorter than 3 bp) insertions and deletions at mono/polynucleotide repeats. In our study in COSMIC V2 (Table 3), only 8.4% of cases (9/107 – W65924, W373, W562, W555, W370, W216, W341, W99125, W229) with signature 6 showed high numbers of InDels (range from 144 to 445) (Additional file 3). In contrast, most GC cases carrying signature 6 (91.6%) had only a few InDels (range from 0 to 72).

We performed cluster analysis for the 43 and 21 “NoMapping” GC cases based on their 96 different mutated trinucleotide patterns from “ALL” and “AA”, separately. We found that both could be divided into three clusters, but given the small sample sizes, no additional analyses of these clusters were performed (data not shown).

### COSMIC V2 mutational signatures using GLM in GC subtypes

We also investigated mutational signatures in three separate subtypes of GC defined by tumor location, histology and grade, and survival with COSMIC mutational signatures V2 using GLM. There were no significant differences between the groups (data not shown).

## Discussion

GC is a highly molecularly heterogeneous disease. More than half of all GC cases worldwide occur in East Asia, predominantly in China. Since no previous study has reported on the full distribution of mutational signatures in Chinese GC patients, we used WES of matched tumor-normal pairs from 175 GC patients to investigate mutational signatures in Chinese GC cases.

Our evaluation for significantly mutated genes identified six such genes (*B2M*, *ELF3*,* RHOA*,* RPL22*,* TP53*, *ARID1A)* that were present in at least 2% of patients; only the somatic mutations in *TP53* and *ARID1A* were found in more than 10% of patients. *TP53* and *ARID1A* were also the most frequently mutated genes in the TCGA samples (both White and Asian). The other four genes (*B2M*, *ELF3*,* RHOA*,* RPL22)* were mutated in the TCGA samples, albeit at low frequencies, similar to what was observed in the Chinese GC patients. *TP53*, *ARID1A*, and *RHOA* have previously been significantly associated with GC tumors [9, 10, 27]. Mutations in *TP53* have consistently been the most frequently observed alteration in the literature in GCs and they were also the most frequent in the current study. *B2M*, *ELF3*, and *RPL22* have each been previously reported in GC tumors [5, 14]. Prior studies reported different genes in GC tumors with high (i.e., hypermutated) numbers of somatic alterations compared to GC tumors with low mutation burdens. The current study had few tumors with high numbers of somatic alterations (i.e., 11 tumors had > 300 somatic alterations). To determine the potential influence of these hypermutated tumors on our results, we re-evaluated mutated genes using MutsigCV after excluding these 11 tumors and determined that four genes from our full analysis (*TP53*, *ARID1A*, *ELF3*, *B2M*) remained significant (q < 0.01). Further, three additional genes (*DDX25*,* RPL4*,* PHLDA1*) had q < 0.01, although their alterations each occurred in less than 2% of cases. None of these three genes have previously been associated with GC, although *PHLDA1* showed decreased protein expression in GC by immunohistochemistry [54]. Also, although *RHOA* was no longer significant, it was the ninth ranked gene (ranked by *P*-value with *P* = 0.00009 and q = 0.19) in this additional analysis.

Using COSMIC V3 mutation signatures in the 175 Chinese GC cases, we identified 14 SBS signatures with cutoff at ≥ 0.57%, one DBS signature that did not match any previously described DBS signatures, and three ID signatures (A, B, C), of which only signature A matched a previously identified signature (ID2) [37]. Comparison of 254 White TCGA GC cases with 256 Asian GC cases (81 from TCGA and 175 Chinese from the current study) showed that the two groups shared the same most frequent SBS signatures (SBS01, SBS05, SBS15, SBS20, and SBS40) but the frequencies of SBS01 and SBS20 were significantly higher in the 254 White GC TCGA cases compared to the 256 Asian GC cases. As widely acknowledged, particularly in the COSMIC mutational signature database [37, 39], SBS01 represents an endogenous mutational process stemming from the spontaneous or enzymatic deamination of 5-methylcytosine to thymine, resulting in G:T mismatches in double-stranded DNA. This signature embodies a clock-like mutation pattern, where the number of mutations correlates with the age of the individual in most cancers and normal cells. There was no significant difference in the age at diagnosis between the two groups in this study (data not shown) which is not surprising given that exome sequencing data has limited power to detect SBS01 mutations and there are many other factors that may influence this association (e.g. tumor purity, copy number, clonality).

Regarding SBS20, it is associated with defective DNA mismatch repair and microsatellite instability (MSI), commonly observed in stomach and colorectal cancers, potentially enriched with *POLD1* mutations [37, 39]. Figure 1 clearly illustrates that samples with SBS20, in general, exhibit a higher mutational signature burden compared to other samples. The Asian GC cases (*n* = 15) with SBS20 (defined as having ≥ 100 mutations for this signature) were all among the top 30 Asian GC cases by mutation count. Similarly, the 29 White GC cases with SBS20 were all among the top 50 White GC cases by mutation count. Further, we observed that tumor samples from the White GC cases had a significantly higher prevalence of SBS20 than the tumor samples from the two Asian GC groups. In addition, 10 White GC cases harbored somatic mutations in *POLD1*, half of which occurred in cases who had SBS20. In contrast, 6 Asian GC cases had somatic mutations in *POLD1*, only 1 of which had SBS20. Hence, only a small proportion of high tumor mutation burden samples with SBS20 signatures may be attributed to *POLD1* mutations and thus, there may be other mutagenesis forces for SBS20 in GC, particularly for tumors from Asia. Additional studies will be required to address this issue.

There were also several very rare signatures observed in the Asian GC cases which suggests potential heterogeneity between samples or, alternatively, overfitting of models. These issues require additional evaluation in studies with larger sample sizes.

Among the SBS signatures, SBS06 and SBS15 have been proposed to relate to defective DNA mismatch repair and are associated with ID1 and ID2 signatures [37]. ID2, observed in this study (98%, 171/175 cases), like TCGA GC cases (99%, 140/142), has been found in almost all cancer cases investigated to date, and has previously been shown to be highly elevated in cancer samples with defective DNA mismatch repair. Further, this signature has been associated with SBS01 in non-hypermutated samples and SBS15 and SBS06 in mismatch repair samples. SBS01, SBS15, and SBS06 were among the most frequent signatures observed in the current study. Although the ID1 signature has also been associated with defective DNA mismatch repair, it was not observed in the Chinese GC cases. Our detailed evaluation showed that most Chinese GC cases carried multiple SBS signatures, indicative of extensive heterogeneity between tumors.

Mutational signatures in cancer genomes are likely to involve multiple mutational processes. Thus, the catalogue of mutational signatures faces many challenges, including differentiating somatic alterations and developing new analytic methods that are robust even for studies with small sample sizes. COSMIC mutational signatures have continued to evolve (V2 and V3) as new knowledge is gained [37]. With our relatively small sample size, our desire to reduce false positives, and interest in comparing our findings to previous evaluations of GC tumors [17, 26, 27], we chose GLM models to analyze COSMIC signatures (V2) in the 175 Chinese GC cases evaluated here. We observed 12 of the 30 COSMIC V2 signatures previously identified. Signature 6 was the most frequent, followed by signatures 15 and 1 in both “ALL” and “AA”. Petljak and Alexandrov separated mutational signatures into three categories using COSMIC V2: (i) signatures associated with endogenous mutational processes (*n* = 11), (ii) signatures associated with exogenous mutational processes (*n* = 7), and (iii) signatures of mutational processes with unknown origins (*n* = 13) [55]. Signatures 1, 6, and 15 (in V2) are proposed as endogenous mutational signatures. Signature 1 is associated with the activity of endogenous mutational clocks active in normal somatic cells that are present in the normal germline [55, 56].

Signatures 6 and 15 belong to a group (including 6, 15, 20, and 26) believed to result from loss of DNA mismatch repair activity and are often found in the same samples [37]. Signature 6 was recently reported in Chinese GC [26, 27] and is predominantly described by C > T mutations at N*C*G and G*C*N trinucleotides, as well as C > A mutations at C*C*T trinucleotides [55]. Chen et al. [26] used mSignatureDB to evaluate COSMIC V2 mutational signature patterns in cases from three studies, including 74 Chinese GC patients, TCGA samples, and 78 WES Chinese GC samples from a study by Chen et al. [17]. Signatures 1, 6, and 17 were the most frequent signatures observed across the three studies, accounting for most mutational processes. In TCGA, however, signature 15 was seen at a higher frequency than that of signature 17 [26]. Although frequencies differed somewhat across the three studies, signature 6 was the most frequent signature in TCGA and second most frequent signature in Chen et al. (where signature 1 was most frequent), consistent with findings from the current study where signatures 6, 1, and 15 were the top rank ordered signatures. While signature 17 was observed in the current study, it was much less frequent than in Chen et al. [26].

The current study was limited by relatively low sequencing coverage which means we may have missed some potential somatic alterations. Our relatively small sample size also limited our power to incorporate risk factor information (e.g., smoking, alcohol consumption) or clinical characteristics into the examinations of mutational signatures or significantly mutated genes. It was also not possible to evaluate potential therapeutic or prognostic differences in significantly mutated genes or by mutational signatures. For example, Xing et al. reported that signature 18*, which occurred almost exclusively in non-coding regions, showed worse prognosis in diffuse GC compared to other counterparts [27]. Similarly, Alexandrov et al. [33] reported that a subset of GC tumors with COSMIC V2 signature 3 had defective double-strand DNA break repair by homologous recombination, and therefore might benefit from either platinum therapy or PARP inhibitors. Further investigation in studies with larger samples sizes is needed. Also, since our study used WES data, we could not investigate non-coding regions. Finally, we could not evaluate microsatellite instability in tumors or correlate it with observed mutational signatures proposed to be related to DNA mismatch repair.

## Conclusions

In conclusion, our study provides a detailed distribution of mutational signatures based on COSMIC V2 and V3 in 175 Chinese GC cases. The most frequent SBS signatures in our study (SBS05, SBS01, SBS15, SBS20, SBS06) were like those in White GC cases from TCGA. However, we observed numerous rarer SBS signatures not seen in TCGA that may be unique to Chinese GC cases but need to be evaluated further in studies with larger sample size. Also, most GC cases in the current study carried multiple signatures. Finally, COSMIC mutational signature analyses (both V2 and V3) showed extensive heterogeneity that should be examined further to improve our understanding of gastric cancer.

## Electronic supplementary material

Below is the link to the electronic supplementary material.

Supplementary Material 1

Supplementary Material 2

Supplementary Material 3

Supplementary Material 4

Supplementary Material 5

Supplementary Material 6

Supplementary Material 7

Supplementary Material 8

Supplementary Material 9

Supplementary Material 10

Supplementary Material 11

Supplementary Material 12

Supplementary Material 13

Supplementary Material 14

Supplementary Material 15

## Data Availability

Access to whole exome sequencing data can be found at: https://dataview.ncbi.nlm.nih.gov/object/PRJNA702785?reviewer=jsvnkpti6epj03pf7l1o1ugimp.

## Abbreviations

COSMIC: Catalogue of Somatic Mutations in Cancer
DBS: Doublet base substitution
GC: Gastric cancer
ID: InDel or insertion and deletion
SBS: single base substitution
TCGA: The Cancer Genome Atlas
WES: Whole exome sequencing
