# Supplementary material for: Mutational signatures in 175 Chinese gastric cancer patients
Source: BMC Cancer. 2024 Sep 30;24:1208. doi: 10.1186/s12885-024-12968-2 (PMC11440915; doi:10.1186/s12885-024-12968-2)
Supplement: Supplementary file 5 — Supplementary Material 5 [file 12885_2024_12968_MOESM5_ESM.pdf]

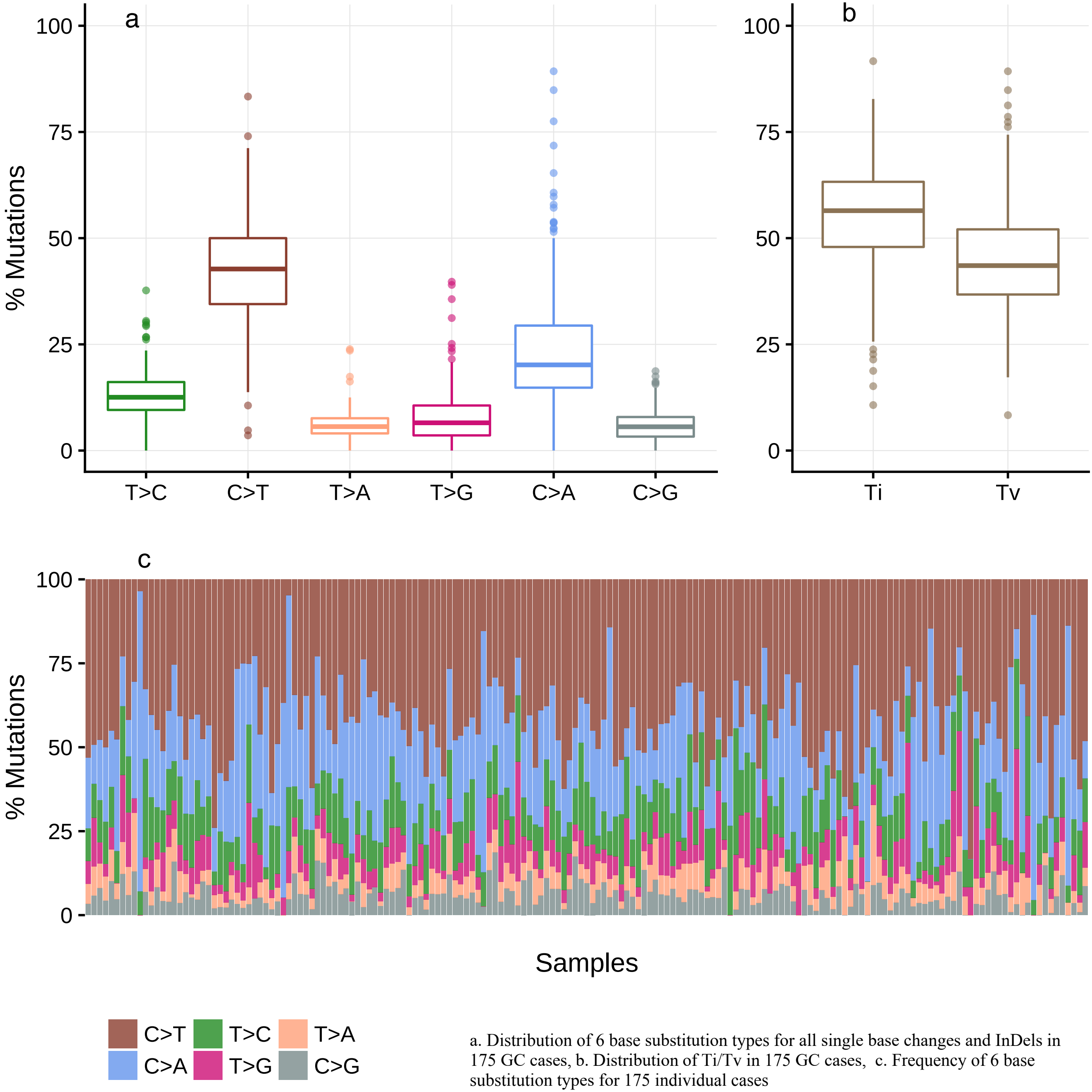

a. Distribution of 6 base substitution types for all single base changes and InDels in 175 GC cases, b. Distribution of Ti/Tv in 175 GC cases, c. Frequency of 6 base substitution types for 175 individual cases
