# Supplementary material for: Mutational signatures in 175 Chinese gastric cancer patients
Source: BMC Cancer. 2024 Sep 30;24:1208. doi: 10.1186/s12885-024-12968-2 (PMC11440915; doi:10.1186/s12885-024-12968-2)
Supplement: Supplementary file 10 — Supplementary Material 10 [file 12885_2024_12968_MOESM10_ESM.pdf]

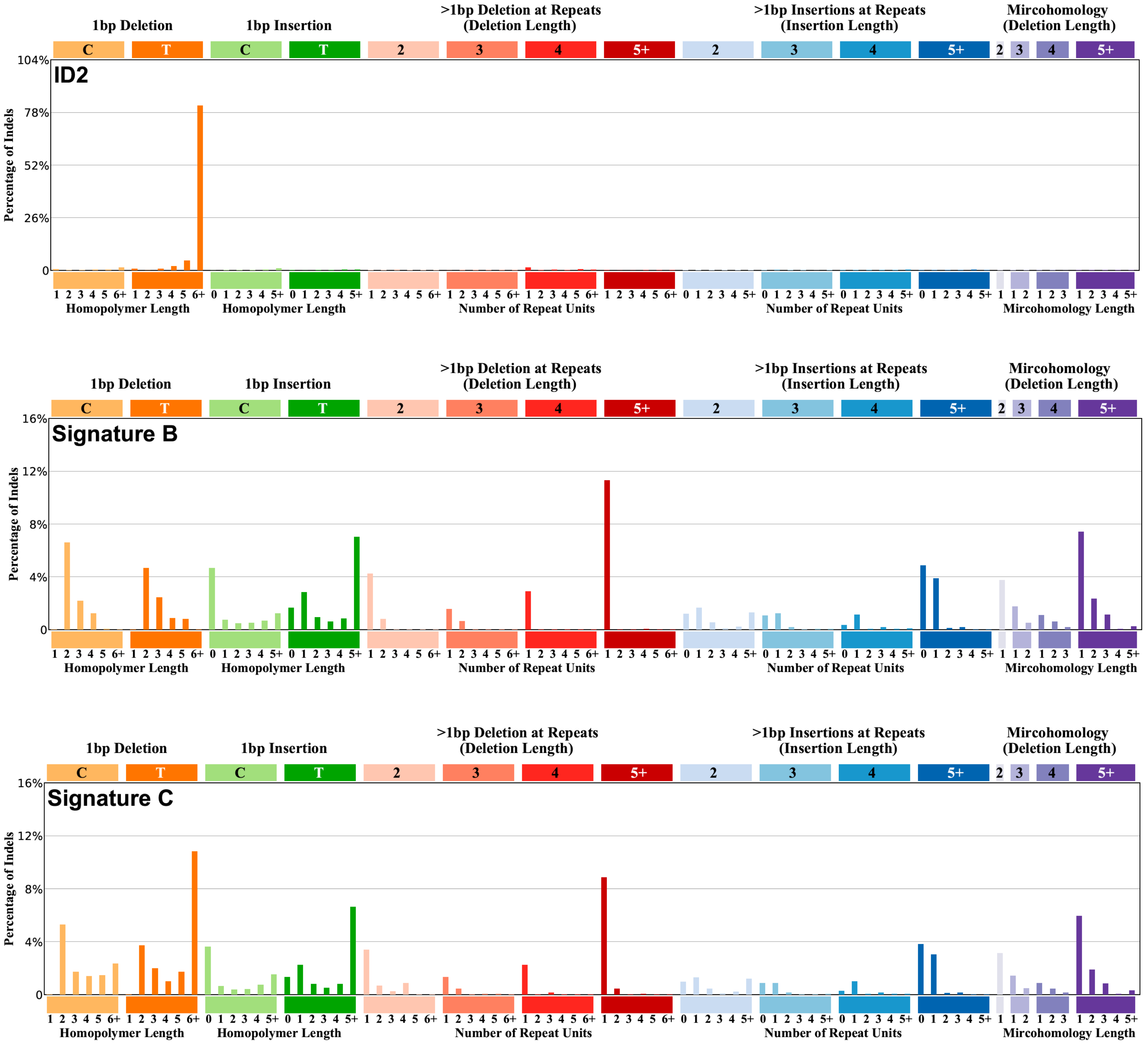

ID mutational signatures/patterns. Three ID patterns were observed (ID2, B, C) but patterns B and C did not match any known COSMIC V3 ID signatures (n=175 GC cases).
