# Supplementary material for: Mutational signatures in 175 Chinese gastric cancer patients
Source: BMC Cancer. 2024 Sep 30;24:1208. doi: 10.1186/s12885-024-12968-2 (PMC11440915; doi:10.1186/s12885-024-12968-2)
Supplement: Supplementary file 12 — Supplementary Material 12 [file 12885_2024_12968_MOESM12_ESM.pdf]

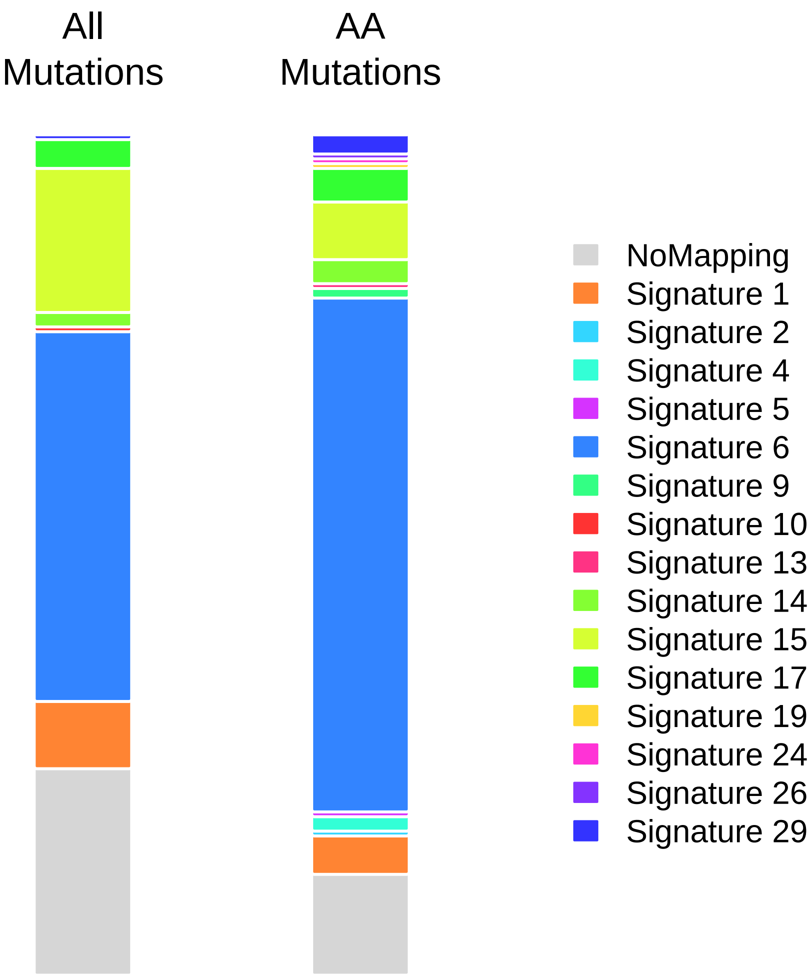

Frequency of COSMIC V2 signatures for all single base changes (“ALL”) and amino acid alterations (“AA”) using GLM model
