# Supplementary material for: Mutational signatures in 175 Chinese gastric cancer patients
Source: BMC Cancer. 2024 Sep 30;24:1208. doi: 10.1186/s12885-024-12968-2 (PMC11440915; doi:10.1186/s12885-024-12968-2)
Supplement: Supplementary file 14 — Supplementary Material 14 [file 12885_2024_12968_MOESM14_ESM.pdf]

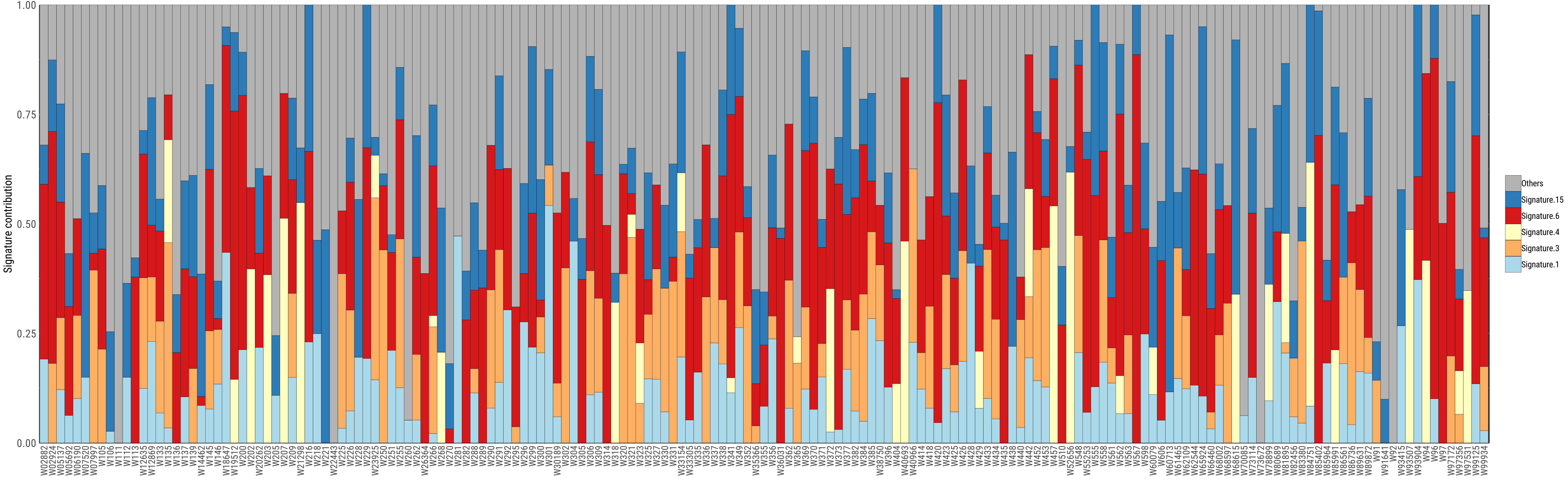

The most significant mutational signatures based on COSMIC V2 and modified deconstructSigs. Signatures 1, 3, 4, 6, 15, and ‘others’ shown (n=175 GC cases).
