# Supplementary material for: Mutational signatures in 175 Chinese gastric cancer patients
Source: BMC Cancer. 2024 Sep 30;24:1208. doi: 10.1186/s12885-024-12968-2 (PMC11440915; doi:10.1186/s12885-024-12968-2)
Supplement: Supplementary file 15 — Supplementary Material 15 [file 12885_2024_12968_MOESM15_ESM.docx]

**Supplementary Information**

**Additional file 1.** Whole exome sequencing coverage statistics (hg19) for GC tumor and matched normal samples

**Additional file 2.** (A) Summary of clinical characteristics (n=175 GC cases)

(B) Individual clinical characteristics (n=175 GC cases)

**Additional file 3.** Type and number of somatic alterations in each GC case (n=175)

**Additional file 4.** Type and number of alterations in 104 mutated genes by MutSigCV analysis (*P*≤ 0.01; n=175 GC cases)

**Additional file 5.** (A) Distribution of 6 base substitution types for all single base changes and InDels in 175 GC cases

(B) Distribution of Ti/Tv in 175 GC cases

(C) Frequency of 6 base substitution types for 175 individual cases

**Additional file 6.** (A) Distribution of 96 trinucleotide changes in 175 GC cases

(B) Summary distribution frequency of 96 trinucleotide changes in 175 GC cases

**Additional file 7.** Status of SBS mutational signatures (COSMIC V3) (175 GC cases)

1. Distribution of SBS mutational signatures in all cases combined (COSMIC V3; n=175 GC cases)

1. Distribution of SBS mutational signatures in each case and percent similarity (COSMIC V3; n=175 GC cases)

**Additional file 8.** Status of DBS in Signature A (COSMIC V3; n=175 GC cases) (Signature A did not fit any known DBS signatures)

1. Number Signature A for DBS in each case
2. Frequency of the 78 classes of DBS in Signature A

**Additional file 9.** Status of ID signature patterns (COSMIC V3; n=171 GC cases)

1. Summary of mutations in ID2 and signatures A, B, & C
2. Number of mutations of Signatures A, B, & C in InDels mutational signatures observed in each case
3. Similarities among Signatures 83-A, 83-B, & 83-C
4. Frequency of the 83 classes of InDels for the 3 ID signature patterns observed [NOTE: Signatures B and C did not fit any known ID signatures].

**Additional file 10.** ID mutational signatures/patterns. Three ID patterns were observed (ID2, B, C) but patterns B and C did not match any known COSMIC V3 ID signatures (n=175 GC cases).

**Additional file 11.** Mutational signatures for cases that mapped to any signature (COSMIC V2; n=132 GC cases)

**Additional file 12.** Frequency of COSMIC V2 signatures for all single base changes (“ALL”) and amino acid alterations (“AA”) using GLM model

**Additional file 13.** Frequency of mutational signatures from COSMIC V2 for 175 GC cases using modified deconstructSig

**Additional file 14.** The most significant mutational signatures based on COSMIC V2 and modified deconstructSigs. Signatures 1, 3, 4, 6, 15, and ‘others’ shown (n=175 GC cases).
